# Tiny Tummies, Big Questions: Unpacking Ultra-Processed Ingredients and Additives in Complementary Foods in the United States

**DOI:** 10.3390/nu18040584

**Published:** 2026-02-11

**Authors:** Elizabeth K. Dunford, Alissa Pries, Mona S. Calvo, Daisy H. Coyle

**Affiliations:** 1 The George Institute for Global Health, University of New South Wales, Sydney, NSW 2031, Australia; dcoyle@georgeinstitute.org.au; 2 Department of Nutrition, Gillings Global School of Public Health, The University of North Carolina at Chapel Hill, Chapel Hill, NC 27599, USA; 3 Independent Consultant; 4 Department of Medicine, Division of Nephrology, Icahn School of Medicine at Mount Sinai, New York, NY 10029, USA; mscalvo55@comcast.net

**Keywords:** ultra-processed foods, complementary foods, additives, infants and young children

## Abstract

**Background/Objectives**: Ultra-processed foods (UPFs) consumption is increasing among infants and young children below three years of age, but to what extent is unclear. Understanding ingredient profiles in commercially produced complementary foods is critical given emerging evidence linking UPF consumption in early life with adverse health trajectories. The objective of this study was to assess the degree of processing and characterize the types of ingredients and additives used in commercial complementary foods available in major US grocery stores. **Methods**: This was a cross-sectional analysis of 651 infant and toddler food products sold by the top 10 largest US grocery stores in 2023. Data were collected from 8 of the 10 stores in Raleigh, North Carolina, and from 2 of the 10 stores online. Ingredients were classified into types and subtypes using Codex Alimentarius and US FDA taxonomies. UPFs were identified using the Nova classification system. The number of UPF ingredients per product and the proportion of products that were considered UPFs were calculated. Mean nutrient levels were compared between UPFs and non-UPFs. Results were examined by category and packaging type. **Results**: 71% of products were classified as UPFs. In addition, additives were present in 71% of products, with flavor enhancers (36%), thickeners (29%), emulsifiers (19%), and colors (19%) being the most common UPF-marker additives used. Ingredient counts varied widely (range 1–56), with snacks containing the most ingredients. Processed fruit and vegetable ingredients were common, while dairy, meat, and legume ingredients were uncommon. UPF products contained higher mean levels of total sugar, added sugar, sodium, and energy density than non-UPF products. Added sugars were present exclusively in UPF products. **Conclusions**: Most US commercial complementary foods are ultra-processed and contain multiple additives. These findings highlight the need for improved labeling and regulatory standards for identifying UPF ingredients and additives to ensure the availability of appropriate and healthy products targeting the youngest consumers.

## 1. Introduction

It is now widely understood that consumption of packaged ultra-processed foods and beverages (UPFs) is increasing both in the United States (US) and globally [1]. A defining feature of UPFs is the presence of cosmetic additives, such as flavor enhancers, colors, and emulsifiers [2]. Recent research showed that the proportion of commercially produced complementary products–foods marketed for children up to 36 months of age-purchased by the US population that contain cosmetic additives such as colors and flavors has increased substantially since the early 2000s, and represented the largest growth in purchases containing additives across all food and beverage categories [3].

In recent years, there has been a huge increase in the availability and variety of commercial complementary foods on the market in the US [4,5]. This trend has occurred in tandem with increased research demonstrating that the consumption of UPFs containing multiple ingredients and specific additive classes poses health risks not only for adults, but also for young children [6,7,8,9]. In adults, UPF consumption is linked with overweight, obesity and cardio-metabolic risks; cancer, type 2 diabetes and cardiovascular diseases; irritable bowel syndrome, depression and frailty conditions [10,11]; as well as premature all-cause mortality [8,12]. In children, research has demonstrated a clear link between UPF consumption and cardio-metabolic conditions [13]. In addition to this, taste preferences and dietary habits formed during early childhood can persist into adulthood, with longitudinal studies clearly demonstrating a positive association between the consumption of UPFs and obesity in young children [14,15]. Little doubt exists that children in the US are being increasingly exposed to these foods, with substantial expansion in the types and quantities sold [16].

Complementary feeding, the introduction of family foods and beverages alongside continued breastfeeding, is a critical phase for child growth and development. During this time, what young children eat contributes not only to nutrient intake but also to the development of eating behaviors and food preferences that can persist later in life. Evidence suggests that early exposure to flavors and textures influences acceptance of foods, including preferences for sweet and salty tastes [17,18]. The growing presence of foods that are classified as UPFs under the Nova framework has raised concerns, as these products are frequently formulated with added sugars, sodium, and cosmetic additives intended to enhance sensory appeal [19]. Consumption of UPFs during complementary feeding may therefore have implications beyond nutrient composition, potentially shaping taste preferences and dietary patterns in ways that favor UPFs high in sugar and sodium.

In response to this, there has been a call to better understand the degree of processing and presence of UPF additive markers in commercial products aimed at infants and young children below 36 months of age [20]. Despite research suggesting a negative association between some food additives and adverse health effects in young children [21], there is currently no research examining the extent to which food additives are used in the manufacturing of commercially produced complementary foods in the US. Although prior research has assessed the degree of processing among commercially produced complementary foods available in the European market [22,23,24] and South East Asian market, comparable data from the US are lacking. Given the explosion of UPF availability and consumption in the US, coupled with the understanding that UPFs can have detrimental effects on infants and young children, the objective of this study was to examine the extent to which commercially produced complementary foods available in US grocery stores are UPFs, with an in-depth look into the types of additives and other ingredients used in these products.

## 2. Materials and Methods

### 2.1. Data Source

The dataset for analysis comprised *n* = 651 commercial infant and toddler food products collected in the US in 2023. Data collection methodology has been described previously [16], but in brief, researchers visited one location for eight of the top 10 grocery store chains in the US (Walmart, Kroger, Costco, Ahold Delhaize, Publix, Sam’s Club, Target, and Aldi) in Raleigh, North Carolina, between March and May 2023. Two of the top ten grocery store retailer locations were not located in North Carolina (H-E-B and Safeway), so data were collected from websites for these retailers. Photos of all available products in the “baby” aisle (in-store) or under the “baby” tab (online) were collected. The George Institute’s FoodSwitch content management system was used to enter data captured from product photos [25]. The information extracted from FoodSwitch for analysis included manufacturer name, brand name, product description, ingredients, packaging type and nutrient information per 100 g (energy, protein, total fat, saturated fat, total sugars, added sugars, sodium). For products with data collected from online sources, information for these products was added to the FoodSwitch data extract to ensure the same information was captured.

### 2.2. Food Categorization

Products were categorized using the World Health Organization’s Nutrient and Promotion Profile Model taxonomy [26], with each product placed in one of eight broad food categories: (1) Dry cereals and starches; (2) Dairy foods; (3) Fruit and vegetable purées/smoothies and fruit desserts; (4) Savory meals/meal components: combinations of starches, vegetables, dairy, and/or traditional proteins; (5) Snacks and finger foods; (6) Ingredients; (7) Confectionery; and (8) Drinks. Further subcategory levels can be seen in Table 1. Products were also placed into one of four packaging type categories (Full-size package; Snack-size package; RTE jar/tub/container; Pouch) based on previous research [16].

### 2.3. Ingredient Identification

Ingredients were disaggregated using delimiters and compound ingredient information was extracted when present. Compound ingredients are ingredients that contain two or more sub-ingredients (for example, cheese [pasteurized milk, cultures]). For ingredients with a variety of common names, a master ingredient name was identified (fruit juice concentrate became concentrated fruit juice; Table S1). When a term relating to an agricultural technique was identified (e.g., organic) it was removed. All final classifications were manually checked by the research team.

### 2.4. Categorization of Ingredients

The United States Department of Agriculture (USDA) ingredient category thesaurus [27] was used as a first step to assign each ingredient to a high-level “ingredient type”. Each ingredient was then assigned an ingredient subtype. For additive classifications, these subtypes were based the FDA’s Substances Added to Food inventory, previously known as Everything Added to Foods in the United States [28]. These additive subtypes were then linked to Codex food additive functional classes to facilitate UPF identification (due to differences in Codex and FDA additive class definitions), with this mapping displayed in Table S2. When a specific additive had multiple functions, it was classified under each function. For all remaining categories, taxonomy developed by Gaines et al. [29] was used to enable a deeper look into specific ingredient types. Ten ingredients fell under multiple ingredient type categories. In these cases each product was examined individually to determine the use of the ingredient in each case. All ingredients and their type/subtype allocations can be seen in Table S1.

### 2.5. Identification of Ultra-Processed Products

**Figure 1:**
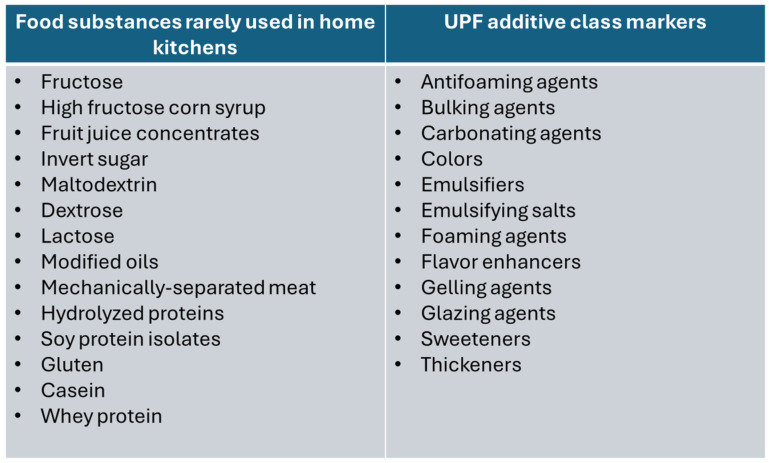
Ingredients used to identify UPFs [2].

The Nova classification system was used to identify products that were considered ultra-processed. The list of substances never or rarely used in the kitchen (see Figure 1) and the 12 Codex additive classes specified to be UPF markers under Nova (antifoaming agents, bulking agents, carbonating agents, colors, emulsifiers, emulsifying salts, foaming agents, flavors/flavor enhancers, gelling agents, glazing agents, sweeteners, thickeners) [2] were used to identify UPFs. If none of these substances or additives were found in the ingredients list, the food was considered not to be ultra-processed.

### 2.6. Statistical Analysis

Statistical analyses were undertaken using Stata V18. The total number of ingredients and compound ingredients in each product was calculated. The total number of ingredients per ingredient type and subtype were also calculated. The mean and range number of ingredients and compound ingredients were examined by category and by packaging type. The proportion of products containing each ingredient type and subtype was examined overall, by category and by packaging type. The proportion of products, by category and packaging type, that were considered UPFs was calculated. The total number of UPF ingredients per product was also calculated. Differences in mean levels of energy (kJ/100 g), total sugar (g/100 g), added sugar (g/100 g), sodium (mg/100 g), protein (g/100 g) and saturated fat (g/100 g) between UPFs and non-UPFs were examined overall and by category. Significant differences were evaluated using Student’s *t*-test, with a significance level of <0.05.

## 3. Results

### 3.1. Number of Ingredients Used in US Commercially Produced Complementary Foods

**Figure 2:**
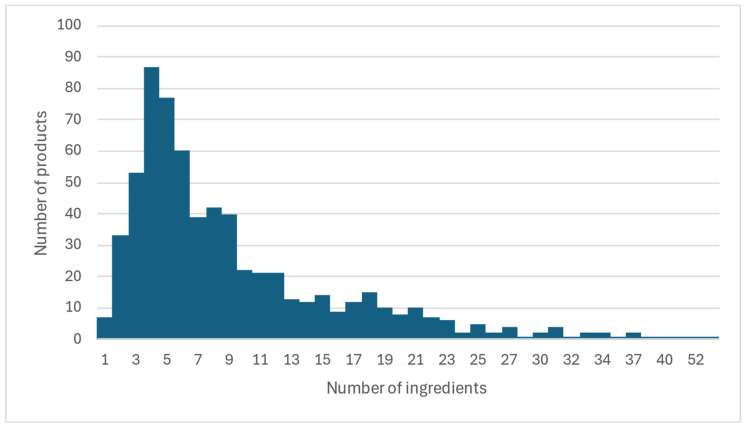
Number of ingredients per product in US commercial complementary foods.

Among the *n* = 651 commercially produced complementary food products examined, the number of ingredients ranged from 1 to 56, with a mean of 9 (Table 1). While only 1% of products contained a single ingredient (*n* = 7), 9% (*n* = 56) had more than 20 ingredients (Figure 2). Ingredient counts varied substantially by category. Fruit-containing products (*n* = 359) had a mean of 6 ingredients (range 1–26), while vegetable-only products (*n* = 48) had a mean of only 3 (range 1–6). Savory meals showed wide variability: products with cheese but no protein showed the highest mean number of ingredients (31; range 16–56), while protein-only meals had a mean of just 2 ingredients. Snacks and finger foods (*n* = 122) had a mean of 16 ingredients (range 3–43) and Confectionery items (*n* = 31) had a mean of 11 ingredients (range = 3–24). Differences were also observed by packaging type (Table S3). Products in snack-size packages (*n* = 31) had the highest mean number of ingredients, (22; range 5–43), followed by full-size packages (mean 13, range 3–27). Ready-to-eat jars/tubs/containers (*n* = 167) averaged 8 ingredients (range 1–56), while pouches (*n* = 308) had the fewest, averaging 7 ingredients (range 2–23).

### 3.2. Types of Ingredients Used in US Commercial Complementary Foods

Additives was the most used type of ingredient, present in 71% of products, followed by fruit (69%) and vegetables (52%; Table 2). Savory meals had the lowest proportion of additives (26%) with all other categories having a minimum of 70% of products containing additives. Fungi, seafood and algae ingredients had the lowest prevalence in baby foods (0%, 0% and 1% respectively) with dairy ingredients present in 17% of products, meat present in 7% of products and nuts/seeds present in 8% of products.

There were 622 unique ingredients found in the overall dataset. Fruit was the ingredient category with the largest number of unique ingredients (*n* = 112) followed by vegetables (*n* = 108) and additives (*n* = 105; Table S4). For fruit, “processed fruit” was the subcategory with the largest number of unique ingredients (*n* = 45) compared to *n* = 23 for “fruit juice” and “dried fruit” and *n* = 21 for “fresh fruit”. Similarly, “processed vegetables” was the vegetable subcategory with the largest number of unique ingredients (*n* = 50). Flavor enhancers were the additive type with the largest number of unique ingredients (*n* = 44) followed by stabilizers (*n* = 27) and colors (*n* = 24).

### 3.3. Ultra-Processed Commercially Produced Complementary Foods

**Figure 3:**
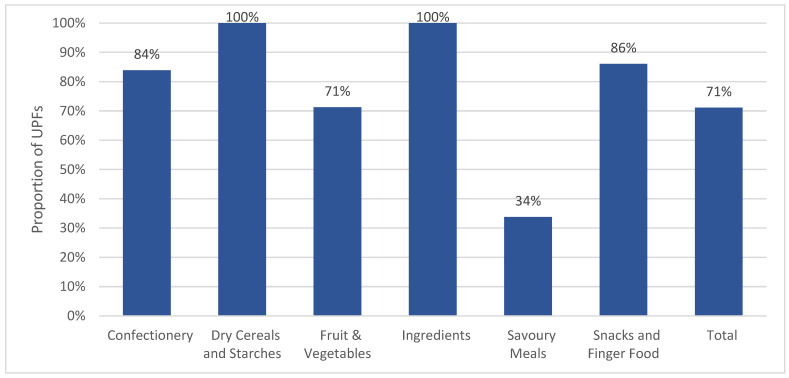
Proportion of US commercial complementary foods considered ultra-processed, by category.

Overall, 71% of all US commercially produced complementary foods were considered ultra-processed using the Nova classification system (Figure 3), ranging from 34% of savory meals to 100% of ingredients and dry cereal and starches. Four out of the six food categories had at least 80% of products considered UPFs. Snack-size packages had the highest proportion of UPFs when examining results by packaging type (94%) followed by full-size packages (86%) and pouches (73%; Figure S1). The number of individual UPF markers per product ranged from 0 to 12 (mean = 2), with 29% of products containing three or more UPF ingredients (Table 3). More than half (56%) of dry cereals and starches contained five or more UPF ingredients, exceeding confectionery with 48%.

Overall, the most common UPF additive classes used were flavor enhancers (36%), thickeners (29%), emulsifiers (19%), and colors (19%; Table S5). Emulsifying salts, gelling agents, glazing agents, bulking agents, foaming agents and NNS were detected infrequently (≤6% each). Confectionery items commonly contained flavor enhancers (78%), colors (58%), and glazing or gelling agents (36%). Dry cereals and starches were dominated by thickeners (88%), emulsifying salts (81%), and emulsifiers (81%), with colors also prevalent (50%). Fruit and vegetable purees, smoothies, and desserts exhibited very low prevalence of UPF additive classes compared to other categories, with only 7% containing colors and 24% containing flavor enhancers. Savory meals showed modest additive use, with thickeners most common (23%) followed by flavor enhancers (16%) and emulsifiers (15%). Snacks and finger foods had the broadest use of UPF additive classes, with high prevalence of flavor enhancers (70%), colors (47%), thickeners (62%), and emulsifiers (38%). Of note is that other additive classes that are not considered markers of ultra-processing were also commonly used in US baby foods. For example, 57% of products contained acidity regulators, 31% contained antioxidants, and 27% contained stabilizers. NNS, foaming agents and sequestrants were relatively uncommon (<1%).

### 3.4. Differences in Nutrient Content Between UPFs and Non-UPFs

Across categories, UPF commercially produced complementary foods consistently exhibited less favorable nutrient profiles than non-UPFs, particularly for sugars, sodium, and energy density. Mean total sugar content was substantially higher in UPF complementary foods compared with non-UPF equivalents (14.0 g/100 g vs. 7.3 g/100 g) (Table S6). Significant differences in total sugar were observed in fruit and vegetable purees/smoothies (10.8 g in UPFs compared to 7.8 in non-UPFs; *p* < 0.0001), and snacks and finger foods (14.4 g/100 g vs. 5.6 g/100 g; *p* = 0.01). While non-UPF fruit and vegetable products contained naturally occurring sugars, their mean values were lower and distributions narrower than those observed among UPFs. Added sugars were exclusively present in UPF products (Table S6).

Mean sodium content was significantly higher among UPF commercially produced complementary foods compared to non-UPFs overall (70 mg/100 g vs. 41 mg/100 g; *p* = 0.004; Table S6), with UPFs also exhibiting a wider range (0–929 mg/100 g vs. 0–714 mg/100 g). Non-UPFs on the other hand generally had minimal sodium levels, often approaching zero. In savory meals, UPFs contained more than double the sodium of non-UPFs (110 vs. 42 mg/100 g; *p* < 0.0001). Sodium variability was particularly large among UPF snacks and finger foods, ranging from 0 to 929 mg/100 g.

Energy density followed a similar pattern, with UPFs exhibiting higher mean energy content than non-UPF products in most food categories (Table S6). This pattern reflects the combined influence of added sugars and refined ingredients in UPF formulations.

In contrast, protein content showed relatively small differences between UPF and non-UPF products across categories, and saturated fat levels were generally low in both groups, with limited between-group variation (Table S6).

## 4. Discussion

This study provides the first comprehensive overview of use of additives and other ingredients in commercially produced complementary foods available in US grocery stores. Out of the 651 products examined, 71% were classified as ultra-processed, with some products containing up to 56 unique ingredients, and in some cases, containing up to 12 Codex UPF ingredient markers. Additives were present in 71% of all products. The most common UPF additive classes used were flavor enhancers, thickeners, emulsifiers, and colors, while acidity regulators, antioxidants, and stabilizers (additives other than the 12 UPF additive marker classes) were also widely used. Fruit (69%) and vegetable (52%) ingredients were also common, but were predominantly included in processed forms. UPFs were associated with poorer nutritional profiles than non-UPF products, including higher sugar and sodium levels and greater energy density, even within food types commonly perceived as suitable for young children, such as fruit-based products. Together, these findings provide a more complete understanding of the processing landscape than previous US-based analyses, which have largely relied on broad product categories or purchasing data rather than ingredient-level classification.

These findings extend prior research which has documented substantial growth in availability of infant and toddler foods containing additives, and which also indicated that baby foods was the category experiencing the largest growth of products containing three or more additives [3]. In addition, recent research has shown US consumers are shifting toward more convenient infant and toddler food products, with sales of pouches having grown nearly 900% since 2010 [16]. Research on commercially produced complementary food products from Europe [22,23,24] and Southeast Asia have highlighted similar concerns about the availability of UPFs and high use of UPF additive classes in this sector. However, our findings suggest that US products have a higher use of these additives and a greater proportion are UPFs than those reported in other countries. Notably, 100% of dry cereals and starches in the present study were classified as UPFs (as compared to only 57% of products available in Southeast Asia), and over half contained five or more UPF ingredient markers.

The results also reinforce global concerns that commercially produced complementary foods increasingly resemble UPFs found outside the baby food aisle, raising questions about how these products fit within dietary guidelines for infants and young children. In Latin America, the Dietary Guidelines for Brazilian Children Under Two Years explicitly advise caregivers to avoid offering UPFs to children under two, and to prioritize foods in their natural or minimally processed forms, underscoring the importance of whole foods for growth and development [30]. While WHO’s complementary feeding guidelines do not yet specifically classify foods by processing level, they stress the necessity of appropriate, safe, and diverse complementary foods and counsel against foods with high levels of sugar, sodium and unhealthy fats, which are characteristic of many UPFs [31].

In addition to extensive use of additives, UPF complementary foods in this study exhibited distinct and less favorable nutrient profiles compared with non-UPF products. Across major food categories, UPFs contained higher mean levels of total sugar, added sugar, sodium, and energy density, with substantially wider ranges, indicating considerable heterogeneity in formulation. Added sugars were present exclusively in UPF products, and sugar levels were notably elevated in categories commonly perceived as healthy, such as fruit- and vegetable-based products. Sodium content was also higher and more variable among UPFs, with some products reaching levels that may contribute meaningfully to daily intakes for young children. In contrast, differences in protein and saturated fat content between UPF and non-UPF products were modest. Collectively, these findings suggest that ultra-processing in US complementary foods is associated not only with complex ingredient profiles but also with nutritional characteristics that conflict with early-life dietary guidance, reinforcing concerns about the suitability of many commercially available products for infants and toddlers.

The dominance of UPF ingredient markers in products aimed at infants and toddlers has notable health implications. Although the long term effects of UPF consumption during infancy are not yet fully understood, growing evidence indicates that early exposure to UPFs can shape taste preferences, dietary habits, and metabolic risk carried into adolescence and adulthood [32]. Importantly, the dietary patterns established in the first years of life tend to persist, and exposure to sweet, uniform textured, and additive-enhanced products may cultivate preferences for highly processed foods later in childhood and adulthood [33]. Flavor enhancers, emulsifiers and colors were common additives used in the products assessed in this study, and the frequent presence of such cosmetic additives warrants attention. These additives are commonly used to improve product stability, texture, and visual appeal, yet there is concern over their inclusion in foods intended for very young children given emerging evidence from experimental and epidemiologic studies. Certain emulsifiers have been linked to alterations in gut microbiota composition and intestinal barrier function, with potential downstream implications for metabolic health [34]. Similarly, exposure to some synthetic color additives has been associated with behavioral outcomes in children, including effects on attention and activity levels [35]. Moreover, the use of thickeners and stabilizers, while legally permitted, warrants careful scrutiny in the context of emerging evidence from animal and mechanistic studies suggesting potential impacts on gut barrier function and inflammatory pathways [36]. Infants and toddlers have uniquely vulnerable gut physiology, making them potentially more sensitive to harmful food components, underscoring the need for research on specific UPF additive classes using age and species appropriate animal models [37]. Research is also needed to identify those additives with multiple functional and cosmetic processing uses that are listed in more than one of the 12 Codex UPF marker additive classes to better focus these research priorities. While the evidence base remains limited for the impact of these additives among infants and young children, the widespread use of such additives in foods marketed for this young age group highlights a need for closer scrutiny of product formulation and its alignment with early childhood dietary guidance.

An additional concern outside the use of UPF marker additives that was highlighted in the current study is the widespread use of processed fruit and vegetable ingredients (compared to fresh ingredients). We found 45 different processed fruit ingredients were used in commercial complementary food products, compared to only 21 fresh fruit ingredients, with fruit juice concentrates and puree concentrates widely used. Although these ingredients do not always meet the regulatory definition of added sugars, many function identically from a metabolic and flavor standpoint. This early sugar exposure in young children may contribute to early preference for sweet tastes and may contribute to growing rates of early childhood obesity [38].

The current US regulations governing baby foods do not explicitly address levels of processing or restrict the use of functional additives. The FDA’s labeling requirements do not differentiate between additive categories that signal ultra-processing (such as flavor enhancers, colors, and emulsifiers) and other additives used for safety. As a result, caregivers lack clear guidance on how to make the most healthful choices when browsing grocery store shelves. In contrast, international bodies such as the WHO and UNICEF have cautioned against marketing infant and toddler foods that contain added sugars, high sodium, or that contain ingredients that enhance sweet taste, color, or texture. The European Union imposes even stricter additive standards for foods marketed to infants and young children, particularly with respect to colors and flavorings.

This study has several strengths. It is the first US-based analysis to systematically characterize ingredient types, additive classes, and UPF classification across commercially produced complementary foods available from major US retailers-critical information for monitoring adverse health outcomes with age. The dataset includes detailed classification of more than 100 additive subtypes and more than 600 individual ingredients. The examination of UPF classification alongside detailed additive categorization offers a multidimensional perspective on ultra-processing. Limitations include reliance on ingredient lists, which do not disclose the quantities of individual ingredients or the use of additives as processing aids or the intensity of processing steps. In addition, although a large representative dataset was used in the analysis, sales data were not available, and so the results should be interpreted in terms of availability rather than purchase or consumption. Nonetheless, these limitations do not materially alter the central finding that UPFs are pervasive across the US baby food market.

## 5. Conclusions

This study characterized the current UPF status of US commercially produced complementary foods based on the ingredient types and additive classes contained in each product, revealing widespread ultra-processing and additive use in most products marketed for infants and toddlers available in grocery stores. Given the central importance of early nutrition for lifelong health, and the growing evidence linking UPFs with adverse health outcomes, efforts to reduce the availability of UPFs and increase transparency in complementary food product formulations should be considered a public health priority. Regulatory action, stronger labeling standards aligned with global dietary guidance for young children, and industry reformulation could play pivotal roles in shaping a food environment that supports healthier dietary trajectories for US infants and young children.

## Figures and Tables

**Table 1:** Number of ingredients and compound ingredients in US commercial complementary foods, by category.

Category/Subcategory		Main Ingredients		Compound Ingredients		Total Ingredients	
	N	Mean (SD)	Range	Mean (SD)	Range	Mean (SD)	Range
Dry cereals/starches	16	15 (6)	(4–25)	1 (2)	(0–6)	16 (7)	(4–27)
Fruit and vegetables							
Fruit-containing	359	6 (3)	(1–24)	0 (1)	(0–6)	6 (4)	(1–26)
Vegetable-containing	48	3 (1)	(1–6)	0 (0)	(0–2)	3 (1)	(1–6)
Savory meals							
No protein/cheese	15	10 (4)	(5–16)	3 (3)	(0–9)	13 (6)	(5–25)
Cheese but no protein	9	23 (8)	(13–37)	8 (6)	(3–19)	31 (12)	(16–56)
Protein not named first	43	9 (4)	(2–24)	5 (8)	(0–31)	14 (10)	(4–52)
Protein named first	4	3 (1)	(3–4)	0 (0)	(0–0)	3 (1)	(3–4)
Protein only	3	2 (0)	(2–2)	0 (0)	(0–0)	2 (0)	(2–2)
Snacks and finger foods	122	13 (6)	(3–36)	2 (3)	(0–19)	16 (8)	(3–43)
Ingredients	1	10 (0)	(10–10)	0 (0)	(0–0)	10 (0)	(10–10)
Confectionery	31	10 (4)	(3–18)	1 (2)	(0–6)	11 (5)	(3–24)
Total	651	8 (6)	(1–37)	1 (3)	(0–31)	9 (8)	(1–56)

**Table 2:** Proportion of ingredient types in US commercial complementary foods.

Category/Subcategory	N	Additives	Algae	Compound Ingredients	Dairy	Eggs	Fats and Oils	Fruits	Fungi
Dry cereals/starches	16	100%	0%	0%	0%	0%	0%	44%	0%
Fruit and vegetables	407	70%	1%	1%	14%	0%	2%	75%	0%
Fruit-containing	359	79%	1%	1%	16%	0%	1%	86%	0%
Vegetable-containing	48	2%	0%	0%	0%	0%	13%	0%	0%
Savory meals	74	26%	0%	19%	19%	7%	66%	24%	1%
No protein/cheese	15	20%	0%	7%	7%	0%	67%	40%	0%
Cheese but no protein	9	89%	0%	56%	100%	56%	78%	11%	11%
Protein not named first	43	16%	0%	19%	9%	0%	74%	26%	0%
Protein named first	4	25%	0%	0%	0%	0%	0%	0%	0%
Protein only	3	0%	0%	0%	0%	0%	0%	0%	0%
Snacks and finger foods	122	93%	2%	11%	15%	5%	58%	70%	1%
Ingredients	1	100%	0%	0%	0%	0%	100%	0%	0%
Confectionery	31	84%	0%	0%	58%	6%	6%	94%	0%
All products	651	71%	1%	5%	17%	2%	20%	69%	<1%
Category/subcategory	Grains	Herbs and spices	Isolated ingredients	Legumes	Meat	Nuts and seeds	Seafood	Vegetables	Water
Dry cereals/starches	100%	0%	94%	19%	0%	0%	0%	13%	0%
Fruit and vegetables	23%	10%	26%	8%	0%	10%	0%	50%	40%
Fruit-containing	25%	11%	28%	7%	0%	11%	0%	44%	35%
Vegetable-containing	6%	4%	8%	21%	0%	0%	0%	90%	77%
Savory meals	59%	55%	11%	35%	61%	1%	1%	86%	92%
No protein/cheese	73%	67%	20%	67%	0%	0%	0%	100%	93%
Cheese but no protein	78%	100%	44%	33%	0%	11%	0%	89%	100%
Protein not named first	51%	44%	2%	30%	88%	0%	2%	95%	95%
Protein named first	100%	0%	0%	0%	100%	0%	0%	0%	100%
Protein only	0%	100%	0%	0%	100%	0%	0%	0%	0%
Snacks and finger foods	93%	63%	63%	16%	0%	11%	0%	48%	13%
Ingredients	0%	100%	100%	0%	100%	0%	0%	100%	100%
Confectionery	10%	6%	61%	0%	0%	3%	0%	29%	6%
All products	41%	25%	34%	13%	7%	8%	<1%	52%	38%

**Table 3:** Number and proportion of UPF ingredient markers used in US commercial complementary foods, by category.

Number of UPF Markers	Confectionery (*n* = 31)	Dry Cereals/ Starches (*n* = 16)	Fruits and Vegetables (*n* = 407)	Ingredients (*n* = 1)	Savory Meals (*n* = 74)	Snacks and Finger Food (*n* = 122)	Total (*n* = 651)
0	5 (16%)	0 (0%)	117 (29%)	0 (0%)	51 (69%)	18 (15%)	191 (29%)
1	2 (6%)	2 (13%)	181 (44%)	0 (0%)	5 (7%)	14 (11%)	204 (31%)
2	0 (0%)	0 (0%)	48 (12%)	0 (0%)	7 (9%)	11 (9%)	66 (10%)
3	3 (10%)	2 (13%)	46 (11%)	0 (0%)	4 (5%)	26 (21%)	81 (12%)
4	6 (19%)	3 (19%)	4 (1%)	1 (100%)	0 (0%)	19 (16%)	33 (5%)
5	1 (3%)	8 (50%)	3 (1%)	0 (0%)	1 (1%)	9 (7%)	22 (3%)
6	6 (19%)	1 (6%)	3 (1%)	0 (0%)	3 (4%)	9 (7%)	22 (3%)
7	5 (16%)	0 (0%)	3 (1%)	0 (0%)	3 (4%)	9 (7%)	20 (3%)
8	3 (10%)	0 (0%)	2 (<1%)	0 (0%)	0 (0%)	4 (3%)	9 (1%)
9	0 (0%)	0 (0%)	0 (0%)	0 (0%)	0 (0%)	1 (1%)	1 (<1%)
10	0 (0%)	0 (0%)	0 (0%)	0 (0%)	0 (0%)	1 (1%)	1 (<1%)
12	0 (0%)	0 (0%)	0 (0%)	0 (0%)	0 (0%)	1 (1%)	1 (<1%)

## Data Availability

Data cannot be shared due to the proprietary nature of the FoodSwitch data.

## Supplementary Materials

The following supporting information can be downloaded at: https://www.mdpi.com/article/10.3390/nu18040584/s1, Figure S1: Proportion of US commercial complementary foods considered ultra-processed, by packaging type; Table S1: Ingredient taxonomy for US commercial complementary foods; Table S2: Mapping of Codex UPF additive classes to FDA technical effect classes; Table S3: Number of ingredients and sub-ingredients in US commercial complementary foods, by packaging type; Table S4: Number of unique ingredients used in US commercial complementary foods; Table S5: Proportion of US commercial complementary foods containing each additive class, both UPF and non-UPF; Table S6 (a) Mean and range sugar content in US commercial complementary foods, by category (b) Mean and range sodium content in US commercial complementary foods, by category (c) Mean and range energy content in US commercial complementary foods, by category (d) Mean and range protein content in US commercial complementary foods, by category (e) Mean and range saturated fat content in US commercial complementary foods, by category (f) Mean and range added sugar content in US commercial complementary foods, by category.
