# Supplementary material for: Tiny Tummies, Big Questions: Unpacking Ultra-Processed Ingredients and Additives in Complementary Foods in the United States
Source: Nutrients. 2026 Feb 11;18(4):584. doi: 10.3390/nu18040584 (PMC12942989; doi:10.3390/nu18040584)
Supplement: Supplementary file 1 [file nutrients-18-00584-s001.zip › nutrients-4131945-supplementary.pdf]

Supplementary Material

Title: Tiny Tummies, Big Questions: Unpacking Ultra-processed In-gredients in Complementary Foods in the United States

Table S1: Ingredient taxonomy for US commercial complementary foods

Subtype	Ingredient name
**ADDITIVES**	
**Acidity regulator**	Acetic acid; ammonium bicarbonate; ascorbic acid; calcium carbonate; calcium citrate malate; calcium phosphate; citric acid; disodium phosphate; lactic acid; lemon extract; lemon juice; lemon juice concentrate; lime juice concentrate; magnesium oxide; potassium bicarbonate; potassium chloride; potassium phosphate; sodium bicarbonate; sodium phosphate; tricalcium phosphate
**Anti-caking agent**	Calcium carbonate; calcium phosphate; carrageenan; cellulose gel; cellulose gum; cellulose powder; corn maltodextrin; dextrose; gelatin; guar gum; magnesium oxide; maltodextrin; modified cornstarch; silicon dioxide; soy lecithin; sunflower lecithin; tapioca maltodextrin; tricalcium phosphate; xanthan gum
**Antioxidant**	Ascorbic acid; calcium citrate malate; citric acid; deoiled sunflower lecithin; mixed tocopherols; rosemary extract; silicon dioxide; soy lecithin; sunflower lecithin; vitamin E
**Bulking agent**	Acacia gum; carrageenan; cellulose gel; cellulose gum; cellulose powder; gum arabic
**Carrier**	Acacia gum; carrageenan; gelatin; gum arabic
**Color**	Annatto extract; beet juice color; beet juice concentrate; black carrot extract; blueberry juice; calcium carbonate; caramel color; carrot juice; dried beet juice; fruit and vegetable juice color; fruit juice for color; hibiscus juice; paprika extract color; pumpkin juice concentrate; purple carrot extract color; radish juice; red beet juice concentrate; red beet juice powder; red cabbage extract; red cabbage juice; riboflavin; turmeric extract color; vegetable juice; yellow carrot juice concentrate
**Color retention**	Citric acid
**Emulsifier**	Acacia gum; carrageenan; citrus pectin; disodium phosphate; fruit pectin; gelatin; glycerin; guar gum; gum arabic; lactic acid esters of mono- and diglycerides; pectin; potassium phosphate; sodium acid pyrophosphate; sodium phosphate; soy lecithin; sunflower lecithin; tricalcium phosphate; vegetable glycerin; xanthan gum
**Emulsifying salt**	Disodium phosphate; tricalcium phosphate
**Firming agent**	Calcium carbonate; calcium citrate malate; citrus pectin; fruit pectin; guar gum; pectin; potassium chloride; tricalcium phosphate
**Flavor enhancer**	Acacia gum; acetic acid; alpha amylase; ascorbic acid; autolyzed yeast extract; apple flavor; banana flavor; barley malt extract; black pepper flavor; blueberry flavor; butter flavor; carrageenan; cherry flavor; citric acid; citrus pectin; dextrose; dry

	yeast; flavorings; flavors; french vanilla flavor; fruit pectin; garlic flavor; guar gum; gum arabic; lactic acid; lactose; magnesium oxide; malt extract; mango flavor; mixed berry flavor; natural flavors; peach flavor; pectin; potassium chloride; raspberry flavor; riboflavin; sodium bicarbonate; strawberry extract; strawberry flavor; vanilla; vanilla extract; vanillin; wheat starch; whey
**Flour treatment agent**	Alpha amylase; calcium carbonate; sodium acid pyrophosphate; soy lecithin; sunflower lecithin; tricalcium phosphate
**Foaming agent**	Xanthan gum
**Gelling agent**	Carrageenan; citrus pectin; fruit pectin; pectin
**Glazing agent**	Acacia gum; carrageenan; citrus pectin; fruit pectin; gum arabic; pectin
**Humectant**	Calcium carbonate; calcium phosphate; carrageenan; dextrose; disodium phosphate; gelatin; guar gum; magnesium oxide; modified cornstarch; potassium phosphate; soy lecithin; sunflower lecithin; tapioca starch; tricalcium phosphate
**NNS**	Monk fruit concentrate
**Preservative**	Acetic acid
**Raising agent**	Alpha amylase; ammonium bicarbonate; calcium carbonate; dry yeast; monocalcium phosphate; potassium bicarbonate; sodium acid pyrophosphate; sodium bicarbonate; tricalcium phosphate
**Sequestrant**	Ascorbic acid; biotin; calcium citrate malate; citric acid; disodium phosphate; lactic acid esters of mono- and diglycerides; potassium phosphate; sodium acid pyrophosphate
**Stabilizer**	Acacia gum; ascorbic acid; calcium carbonate; calcium citrate malate; calcium phosphate; carrageenan; citrus pectin; corn maltodextrin; dextrose; disodium phosphate; fruit pectin; gelatin; guar gum; gum arabic; lactic acid esters of mono- and diglycerides; maltodextrin; modified cornstarch; pectin; potassium chloride; potassium phosphate; potassium salt; tapioca fiber; tapioca maltodextrin; tricalcium phosphate; wheat starch; whey; xanthan gum
**Thickener**	Acacia gum; ascorbic acid; carrageenan; citrus pectin; corn maltodextrin; dextrose; disodium phosphate; fruit pectin; guar gum; gum arabic; maltodextrin; modified cornstarch; pectin; potassium chloride; potassium salt; tapioca fiber; tapioca maltodextrin; tricalcium phosphate; wheat starch; whey; xanthan gum
**ALGAE**	
	Chlorella; kelp; spirulina
**COMPOUND INGREDIENTS**	
	Apple filling; bolognese sauce; breadcrumbs; brown rice crisps; cooked turkey pattie crumble; cracker meal; dark chocolate chips; dijon mustard; egg noodles; gravy; leavening; macaroni; pasta; pea protein powder; red lentil pasta; rice crisps; rice protein powder
**DAIRY AND DAIRY PRODUCTS**	
**Cheese**	Cheddar cheese; cheese powder; dried cheddar cheese; mozzarella cheese; parmesan cheese; ricotta cheese; romano cheese

**Dairy alternative**	Coconut kernel extract; coconut milk
**Milk or cream**	Buttermilk; cream; cultured cream; cultured grade a milk; cultured grade a reduced fat milk; cultured lowfat milk; cultured milk; cultured nonfat milk; cultured part skim milk; cultured pasteurized milk; cultured pasteurized part-skim milk; cultured reduced fat milk; cultured whole milk; grade a skim milk; milk; milkfat; nonfat dairy milk; nonfat dry milk; nonfat milk; nonfat milk whey; sour cream solids; whole milk
**Milk powder**	Nonfat milk powder; skim milk powder; sour cream powder; whey powder
**Yogurt**	Dried nonfat milk yogurt; dry yogurt; lowfat yogurt; nonfat greek yogurt; nonfat yogurt; whole milk greek yogurt; whole milk yogurt; yogurt; yogurt powder
**EGGS AND EGG PRODUCTS**	
	Dried egg whites; egg white; egg whites; egg yolks; eggs
**FATS AND OILS**	
**Butter**	Butter; butter fat; unsalted butter
**Fats**	Palm shortening
**Oils**	Avocado oil; black pepper oil; canola oil; chicken fat; coconut oil; extra virgin avocado oil; extra virgin olive oil; garlic oil; high oleic sunflower oil; lemon oil; olive oil; onion oil; palm oil; peanut oil; soybean oil; sunflower and/or canola oil; sunflower and/or safflower oil; sunflower oil; sustainable palm oil; vegetable oil
**FRUITS AND FRUIT PRODUCTS**	
**Dried fruit**	Crushed dates; date paste; dates; dehydrated apples; dried apple juice; dried apple puree; dried apples; dried banana puree; dried bananas; dried blueberries; dried mango; dried peach puree; dried pineapple; dried prunes; dried raspberries; dried raspberry puree; dried strawberries; evaporated apples; prune paste; prune puree; prunes; raisin paste; raisins
**Fresh fruit**	Apples; apricots; avocado; bananas; blackberries; blueberries; cherries; cranberry; dragon fruit; grapes; guava; kiwi; mangoes; orange; peaches; pears; pineapples; plums; raspberries; strawberries; tomatillos
**Fruit juice**	Apple juice; apple juice concentrate; blackberry juice concentrate; blueberry juice concentrate; cherry juice concentrate; concord grape juice; dried orange juice; elderberry juice concentrate; grape juice concentrate; grapefruit juice; lemon juice powder; orange juice; orange juice concentrate; orange juice powder; pear juice; pear juice concentrate; pineapple juice; pineapple juice concentrate; pomegranate juice concentrate; raspberry juice concentrate; strawberry juice concentrate; white grape juice; white grape juice concentrate
**Processed fruit**	Acai puree; apple flakes; apple juice powder; apple powder; apple puree; apple puree concentrate; apricot puree; avocado puree; banana flakes; banana powder; banana puree concentrate; blackberry puree; blueberry juice powder; blueberry powder; banana puree; blueberry puree; cherry puree; dragon fruit puree; dried blueberry crumble; grape puree; guava puree; kiwi puree; lemon powder; mango powder; mango puree; mango puree concentrate; nectarine puree; orange powder; orange puree; papaya puree; papya powder; passionfruit puree; peach flakes; peach puree; peach puree

	concentrate; pear puree; pear puree concentrate; pineapple puree; plum puree; raspberry powder; raspberry puree; seedless strawberry puree; strawberry flavored bits; strawberry powder; strawberry puree
**FUNGI**	
	Inactive yeast; nutritional yeast; torula yeast; yeast
**GRAINS AND GRAIN PRODUCTS**	
**Flours and starches**	Amaranth; barley flour; bleached wheat flour; brown rice flour; corn flour; cornmeal; cornstarch; crisped rice flour; degermed yellow corn meal; degermed yellow cornmeal; durum wheat flour; durum wheat semolina; european stoneground wheat flour; farro; flour; jasmine rice flour; millet flour; oat flour; pearled barley flour; potato starch; rice flour; rice starch; rye flour; semolina flour; sorghum flour; spelt; wheat flour; white rice flour; whole durum wheat flour; whole wheat durum flour; whole wheat flour; yellow corn; yellow corn flour; yellow cornmeal
**Grains**	Barley flakes; corn; gluten free oatmeal; gluten free oats; grond oat flakes; milled amaranth; milled quinoa; milled wholegrain oats; oat flakes; oatmeal; oats; orzo; pearled barley; puffed quinoa; quinoa; quinoa flour; red quinoa; rice; rolled oats; semolina; sorghum meal; wheat bran; wheat semolina; white rice; whole grain barley; whole grain blend
**ISOLATED INGREDIENTS**	
**Mineral**	Calcium d-pantothenate; choline bitartrate; dha algal oil; iron; zinc gluconate; zinc oxide; zinc sulfate; zinc sulfate monohydrate
**Sweeteners**	Agave syrup; brown rice syrup; brown sugar; cane invert syrup; cane sugar; cane syrup; coconut sugar; dried cane syrup; evaporated cane invert syrup; evaporated cane sugar; evaporated cane syrup; honey; invert cane sugar; invert cane syrup; invert sugar; molasses; powdered sugar; sugar; tapioca fiber syrup; tapioca syrup
**Vitamins**	Dicalcium phosphate; folic acid; vitamin A; vitamin B1; vitamin B12; vitamin B2; vitamin B3; vitamin B5; vitamin B6; vitamin C; vitamin D
**Others**	Acidophilus; agave inulin; baking powder; baking soda; bulgaricus; cheese cultures; chicory root fiber; cultures; enzymes; fructooligosaccharide; inulin; l. Paracasei; milk protein concentrate; moringa; oat fiber; pea fiber; pea protein; pea protein isolate; probiotics; pumpkin seed protein; sunflower seed protein; vital wheat gluten; whey protein concentrate; whey protein isolate; yeast beta glucan; yeast extract; yumi superfood blend
**LEGUMES AND LEGUME PRODUCTS**	
**Legume flours**	Chickpea flour; green lentil flour; lentil flour; pea flour; peanut flour; red lentil flour; soy flour
**Legumes**	Black bean puree; black beans; cannellini beans; chickpea puree; chickpeas; dried navy beans; dried peas; navy bean puree; navy beans; pea powder; pea puree; peanut butter; peas; red kidney beans; red lentils; small white beans; white bean puree; white beans; yellow beans
**MEAT AND MEAT PRODUCTS**	
**Beef**	Beef; grass fed beef; grass fed beef bones

**Chicken**	Chicken; chicken bones; chicken meat; ground chicken
**Turkey**	Ground turkey; turkey; turkey bones
**Other**	Grass fed bison; ham
**NUT AND SEED PRODUCTS**	
**Processed seed**	Almond butter; chocolate liquor; cocoa butter; coconut cream; pumpkin seed butter; sunflower kernel paste; sunflower protein; sunflower seed butter
**Raw seeds**	Chia; chia seeds; coconut; flaxseed; flaxseed protein; ground chia seeds; ground flaxseed; milled chia seeds; milled flaxseed; mustard seeds; pumpkin seeds; sunflower seeds
**SEAFOOD AND SEAFOOD PRODUCTS**	
	Salmon
**SPICES, HERBS AND FLAVORINGS**	
**Dried herb or seasoning**	Black pepper; black peppercorns; cardamom powder; celery spice; cinnamon; cinnamon powder; clove; cream of tartar; cumin; dried basil; dried garlic; dried oregano; dried parsley; garlic powder; ground cinnamon; ground ginger; ground rosemary; ground sage; ground turmeric; ground vanilla bean; nutmeg; oregano; paprika; parsley flakes; smoked paprika; spice; spices; turmeric
**Fresh herb**	Basil; cilantro; coriander; mint; parsley; rosemary; sage; thyme
**Meat broths/stocks**	Beef broth; beef stock; chicken bone broth; chicken broth; chicken stock; dried chicken broth; grass fed beef bone broth; turkey bone broth; turkey broth
**Salt**	Himalayan sea salt; salt
**Vinegar**	Apple cider vinegar; balsamic vinegar; distilled vinegar; vinegar
**VEGETABLES AND VEGETABLE PRODUCTS**	
**Dried vegetables**	Dehydrated potato flakes; dried broccoli; dried butternut squash; dried carrots; dried onions; dried pumpkin; dried spinach; dried sweet potato
**Fresh vegetables**	Asparagus; beets; broccoli; butternut squash; carrot; carrot puree; carrots; cauliflower; celery; garlic; ginger; green beans; green pea; green pepper; kabocha squash; kale; leeks; maitake mushroom; mushrooms; onions; parsnips; pea; potato; potatoes; pumpkin; purple carrots; purple sweet potatoes; red beets; red bell peppers; red peppers; rhubarb; shiitake mushrooms; spinach; squash; sweet corn; sweet potatoes; tomatoes; wheatgrass; white potato; winter squash; yellow bell peppers; zucchini
**Processed vegetables**	Arrowroot flour; beet powder; beet puree; broccoli powder; broccoli puree; butternut squash puree; carrot flakes; carrot powder; cassava flour; cauliflower powder; cauliflower puree; celery puree; corn grits; corn puree; diced tomatoes; green bean puree; green pea powder; green pea puree; kale powder; kale puree; leek powder; mashed potatoes; mushroom

	powder; onion powder; onion puree; parsnip puree; pea flakes; potato flakes; potato flour; pumpkin powder; pumpkin puree; purple carrot powder; purple carrot puree; purple pumpkin puree; red beet puree; red bell pepper puree; roasted chili peppers; rutabaga puree; spinach powder; spinach puree; sun-dried tomato powder; sweet corn puree; sweet pea puree; sweet potato powder; sweet potato puree; tapioca flour; tomato paste; tomato powder; tomato puree; zucchini puree
**Vegetable juice**	Carrot juice concentrate; celery juice concentrate; ginger juice; purple carrot juice concentrate; purple carrot juice powder; purple sweet potato juice powder; red beet juice; tomato juice
**WATER**	
	Purified water; seasoned water; water

<u>Table S2:</u> Mapping of Codex UPF additive classes to FDA technical effect classes ***Sourced from Popkin et al, 2024, Lancet Regional Americas***

**CODEX Functional Class (UPF markers)**	**FDA Technical Effect Classes**
Anti-foaming agent	Surface-active agents
Bulking agent	Stabilizers and thickeners
Colour	Colors and coloring adjuncts
Colour retention agent	Colors and coloring adjuncts
Emulsifier	Emulsifiers and emulsifier salts
Emulsifying salt	Emulsifiers and emulsifier salts
Flavour enhancer	Flavor enhancers + Flavoring agents and adjuvants
Foaming agent	Surface-active agents
Gelling agent	Stabilizers and thickeners
Glazing agent	Surface-finishing agents
Sweetener	Non-nutritive sweeteners
Thickener	Stabilizers and thickeners

Table S3: **Number of ingredients and sub-ingredients in US commercial complementary foods, by packaging type**

Packaging type	N	Main ingredients		Sub-ingredients		Total ingredients	
		Mean (SD)	Range	Mean (SD)	Range	Mean (SD)	Range
Full-size package	145	12 (5)	(3-25)	1 (2)	(0-10)	13 (6)	(3-27)
Pouch	308	6 (3)	(2-17)	0 (1)	(0-13)	7 (3)	(2-23)
RTE jar/tub/container	167	7 (6)	(1-37)	2 (5)	(0-31)	8 (10)	(1-56)
Snack-size package	31	18 (8)	(5-36)	4 (4)	(0-19)	22 (11)	(5-43)
**Total**	**651**	**8 (6)**	**(1-37)**	**1 (3)**	**(0-31)**	**9 (8)**	**(1-56)**

Figure S1: **Proportion of US commercial complementary foods considered ultra-processed, by packaging type**

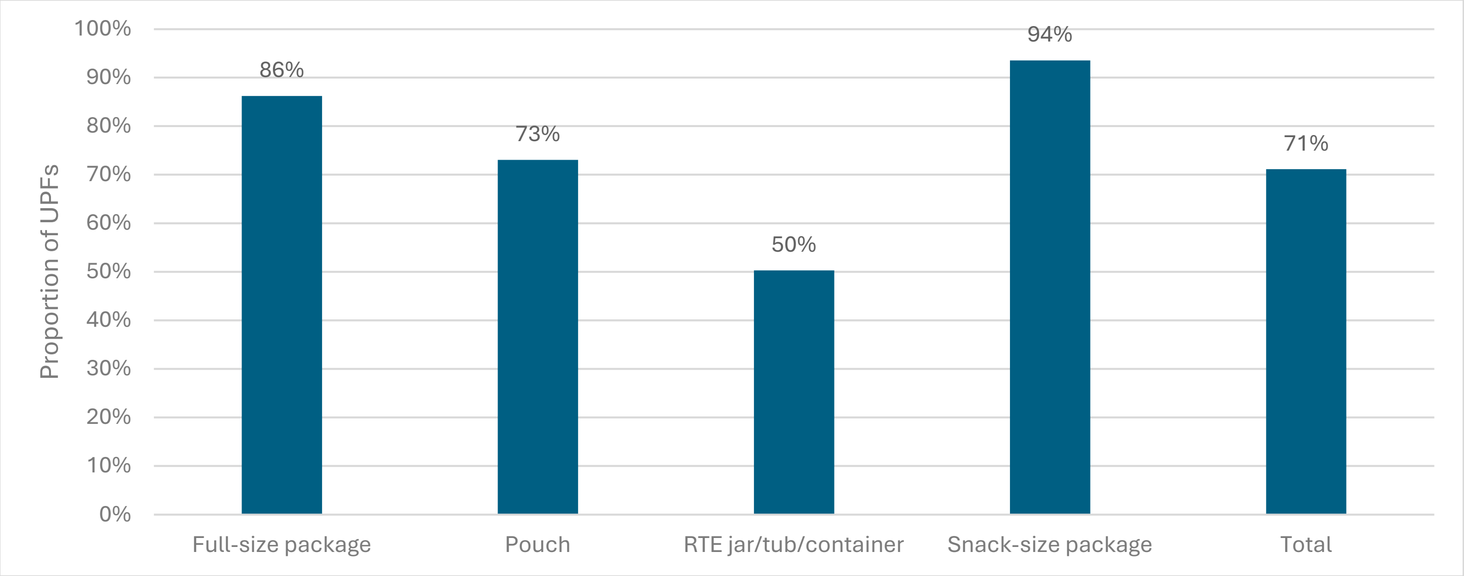

Table S4: **Number of unique ingredients used in US commercial complementary foods**

Ingredient type and subtype	Number of unique ingredients
**Additives**	**105**
ACIDITY REGULATORS	20
ANTICAKING AGENTS	19
ANTIFOAMING AGENTS	0
ANTIOXIDANTS	10
BULKING AGENTS	6
CARBONATING AGENTS	0
CARRIERS	4
COLORS	24
COLOR RETENTION AGENTS	1
EMULSIFIERS	19
EMULSIFYING SALTS	2
FIRMING AGENTS	8
FLAVOR ENHANCERS	44
FLOUR TREATMENT AGENTS	6
FOAMING AGENTS	1
GELLING AGENTS	4
GLAZING AGENTS	6
HUMECTANTS	14
NON-NUTRITIVE SWEETENERS	1
PRESERVATIVES	1
RAISING AGENTS	9
SEQUESTRANTS	8
STABILIZERS	27
THICKENERS	21
**Algae**	**3**
**Compound ingredients**	**17**
**Dairy and Dairy Products**	**45**
CHEESE	7
DAIRY ALTERNATIVE	2
MILK OR CREAM	22
MILK POWDER	5
YOGURT	9
**Eggs and Egg Products**	**5**
**Fats and Oils**	**24**
BUTTER	3
FATS	1
OILS	20
**Fruits and Fruit Products**	**112**
DRIED FRUIT	23
FRESH FRUIT	21
FRUIT JUICE	23

PROCESSED FRUIT	45
**Fungi**	**4**
**Grains and Grain Products**	**60**
FLOURS AND STARCHES	34
GRAINS	26
**Isolated Ingredients**	**66**
SWEETENERS	20
VITAMINS AND MINERALS	19
OTHER	27
**Legumes and Legume Products**	**26**
LEGUME FLOURS	7
LEGUMES	19
**Meats and Meat Products**	**12**
BEEF	3
CHICKEN	4
OTHER	2
TURKEY	3
**Nut and Seed Products**	**20**
PROCESSED SEED	8
RAW SEEDS	12
**Seafood and Seafood Products**	**1**
**Spices, Herbs and Flavorings**	**51**
DRIED HERB OR SEASONING	28
FRESH HERB	8
MEAT BROTHS/STOCKS	9
SALT	2
VINEGAR	4
**Vegetables and Vegetable Products**	**108**
DRIED VEGETABLES	8
FRESH VEGETABLES	42
PROCESSED VEGETABLES	50
VEGETABLE JUICE	8
**Water**	**3**
**Total**	**662**

Table S5: Proportion of US commercial complementary foods containing each additive class, both UPF and non-UPF

UPF additive classes	Confectionery	Dry Cereals/Starches	Fruits/vegetables	Ingredients	Savoury Meals	Snacks/Finger Food	Total
**Total N**	31	16	407	1	74	122	651
**Antifoaming agents**	0%	0%	0%	0%	0%	0%	0%
**Bulking agents**	10%	0%	0%	0%	3%	7%	2%
**Carbonating agents**	0%	0%	0%	0%	0%	0%	0%
**Colors**	58%	50%	7%	0%	11%	47%	19%
**Emulsifiers**	77%	81%	4%	0%	15%	47%	19%
**Emulsifying salts**	0%	81%	1%	0%	5%	17%	6%
**Flavor enhancers**	77%	94%	24%	100%	16%	70%	36%
**Foaming agents**	0%	0%	0%	0%	4%	2%	1%
**Gelling agents**	35%	0%	2%	0%	4%	10%	5%
**Glazing agents**	35%	0%	2%	0%	4%	11%	6%
**Thickeners**	55%	88%	15%	100%	23%	62%	29%
**NNS**	0%	0%	0%	0%	0%	2%	0%
**Non-UPF additive classes**	**Confectionery**	**Dry Cereals/Starches**	**Fruits/vegetables**	**Ingredients**	**Savoury Meals**	**Snacks/Finger Food**	**Total**
**Total N**	31	16	407	1	74	122	651
**Acidity regulators**	42%	88%	68%	100%	18%	42%	57%
**Anticaking agents**	52%	69%	4%	0%	15%	42%	16%
**Antioxidants**	45%	56%	21%	0%	5%	75%	31%
**Carriers**	42%	0%	1%	0%	0%	4%	3%
**Color retention agents**	10%	0%	9%	0%	0%	16%	9%
**Firming agents**	35%	38%	3%	100%	5%	26%	10%
**Flour treatment agents**	0%	63%	2%	0%	7%	36%	10%
**Humectants**	61%	88%	5%	0%	16%	57%	21%
**Preservatives**	0%	0%	0%	0%	1%	0%	0%
**Raising agents**	0%	50%	1%	0%	5%	21%	6%
**Sequestrants**	42%	81%	18%	0%	3%	20%	20%
**Stabilizers**	77%	88%	17%	100%	18%	45%	27%

Table S6 (a) Mean and range sugar content in US commercial complementary foods, by category

**Category/subcategory**	**Total sugar g/100g (UPFs)**			**Total sugar g/100g (non-UPFs)**		
	**N**	**Mean (SD)**	**Range**		**Mean (SD)**	**Range**
Dry cereals/starches	16	12.6 (8.8)	0.0-26.7	0	-	-
Fruit and vegetables	290	10.8 (5.6)	0.0-57.1	117	7.8 (3.6)	1.8-15.0
Savory meals	25	3.0 (2.0)	0.0-8.0	49	2.6 (1.5)	0.0-6.2
Snacks and finger foods	105	14.4 (13.8)	0.0-60.0	17	5.6 (11.1)	0.0-36.4
Ingredients	1	0.0	-	-	-	-
Confectionery	26	59.6 (5.0)	57.1-71.4	5	48.6 (29.6)	0.0-71.4
**Total**	**463**	**14.0 (14.0)**	**0.0-71.4**	**188**	**7.3 (9.5)**	**0.0-71.4**

Table S6 (b) Mean and range sodium content in US commercial complementary foods, by category

**Category/subcategory**	**Sodium mg/100g (UPFs)**			**Sodium mg/100g (non-UPFs)**		
	**N**	**Mean (SD)**	**Range**		**Mean (SD)**	**Range**
Dry cereals/starches	16	42 (36)	0-100	0	-	-
Fruit and vegetables	290	13 (23)	0-286	117	11 (14)	0-84
Savory meals	25	110 (66)	13-200	49	42 (28)	0-117
Snacks and finger foods	105	187 (193)	0-929	17	223 (216)	0-714
Ingredients	1	268	-	-	-	-
Confectionery	26	207 (103)	0-357	5	86 (117)	0-214
**Total**	**463**	**70 (126)**	**0-929**	**188**	**40 (91)**	**0-714**

Table S6 (c) Mean and range energy content in US commercial complementary foods, by category

Category/subcategory	Energy kcal/100g (UPFs)			Energy kcal/100g (non-UPFs)		
	N	Mean (SD)	Range		Mean (SD)	Range
Dry cereals/starches	16	399 (13)	357-429	0	-	-
Fruit and vegetables	290	74 (43)	40-429	117	61 (18)	20-107
Savory meals	25	103 (77)	70-467	49	78 (19)	47-127
Snacks and finger foods	105	401 (50)	286-500	17	422 (70)	357-571
Ingredients	1	141 (0)	-	-	-	-
Confectionery	26	400 (46)	300-429	5	371 (32)	357-429
**Total**	**463**	**179 (158)**	**40-500**	**188**	**106 (115)**	**20-571**

Table S6 (d) Mean and range protein content in US commercial complementary foods, by category

Category/subcategory	Protein g/100g (UPFs)			Protein g/100g (non-UPFs)		
	N	Mean (SD)	Range		Mean (SD)	Range
Dry cereals/starches	16	10.1 (3.6)	6.7-14.3	0	-	-
Fruit and vegetables	290	0.8 (1.9)	0.0-28.6	117	0.9 (0.8)	0.0-2.7
Savory meals	25	3.2 (2.0)	0.9-11.3	49	4.3 (3.0)	1.8-15.5
Snacks and finger foods	105	3.9 (5.5)	0.0-28.6	17	6.6 (9.2)	0.0-28.6
Ingredients	1	12.7 (0)	-	-	-	-
Confectionery	26	12.6 (11.7)	0.0-28.6	5	2.9 (6.4)	0.0-14.3
**Total**	**463**	**2.7 (5.2)**	**0.0-28.6**	**188**	**2.3 (3.8)**	**0.0-28.6**

Table S6 (e) Mean and range saturated fat content in US commercial complementary foods, by category

Category/subcategory	Saturated fat g/100g (UPFs)			Saturated fat g/100g (non-UPFs)		
	N	Mean (SD)	Range		Mean (SD)	Range
Dry cereals/starches	16	0.0 (0.0)	0.0-0.0	0	-	-
Fruit and vegetables	290	0.1 (0.3)	0.0-2.7	117	0.1 (0.3)	0.0-1.5
Savory meals	25	0.8 (0.7)	0.0-2.3	49	0.6 (0.6)	0.0-2.1
Snacks and finger foods	105	0.9 (2.7)	0.0-11.1	17	0.5 (1.3)	0.0-4.4
Ingredients	1	2.1 (0.0)	-	-	-	-
Confectionery	26	0.0 (0.0)	0.0-0.0	5	2.8 (3.9)	0.0-7.1
**Total**	**463**	**0.3 (1.4)**	**0.0-11.1**	**188**	**0.4 (0.9)**	**0.0-7.1**

Table S6 (f) Mean and range added sugar content in US commercial complementary foods, by category

Category/subcategory	Added sugar g/100g (UPFs)			Added sugar g/100g (non-UPFs)		
	N	Mean (SD)	Range		Mean (SD)	Range
Dry cereals/starches	16	2.1 (5.8)	0.0-20.0	0	-	-
Fruit and vegetables	290	0.6 (3.1)	0.0-28.6	117	0.0 (0.0)	-
Savory meals	25	0.2 (0.5)	0.0-1.8	49	0.0 (0.0)	-
Snacks and finger foods	105	9.1 (10.8)	0.0-31.6	17	0.0 (0.0)	-
Ingredients	1	0.0 (0.0)	-	-	-	-
Confectionery	26	26.7 (14.4)	0.0-42.9	5	0.0 (0.0)	-
**Total**	**463**	**4.0 (9.4)**	**0.0-42.9**	**188**	0.0 (0.0)	-
